# Effect of Preoperative nasal iodine Application on Musculoskeletal Surgical Site Infections (SSI)

**DOI:** 10.1017/ash.2025.409

**Published:** 2025-09-24

**Authors:** Anupama Neelakanta, Kristin Fischer, Gebrekidan Mahlet, Jessica Layell, Shelley Kester, Ben Masten, Andrew Ferris, Catherine Passaretti

**Affiliations:** 1 Atrium Health; 2 Atrium Health; 3 Advocate Health - Atrium; 4 Atrium Health; 5 Advocate Health; 6 Atrium Health

## Abstract

**Background:** SSI results in increased mortality, morbidity, length of stay and healthcare costs. Use of nasal iodine for some surgeries has been proposed as an easy, economic alternative to 5-day preoperative chlorhexidine bath and intranasal mupirocin decolonization in SSI prevention but data on effectiveness is limited. We aim to assess the association between preoperative nasal iodine application and odds of SSI. **Methods:** We performed a retrospective study of all total hip replacement, total knee replacement, and spinal fusion surgeries performed between January 2023 through June 2024 in 10 facilities in a large healthcare system. Demographics, clinical risk factors, and procedural data were collated from the electronic health record and merged with SSI data obtained through routine surveillance by trained infection preventionists using standard NHSN (National Healthcare and Safety Network) definitions. Patients with SSI present at the time of surgery were excluded. Nasal iodine compliance was defined as documentation of nasal iodine administration in both nostrils on the day of surgery in the preoperative space. Surgeries where nasal iodine was documented as not given or that had absence of documentation were counted as noncompliant. Descriptive statistics were used to compare compliant and noncompliant patients. Multivariate logistic regression was performed to assess the association between nasal iodine compliance and SSI. **Results:** A total of 14,505 surgeries were included, of which 161 (1.1%) were complicated by SSI. 12,281 (84.6%) of patients were compliant with nasal iodine. Around 55% of the noncompliant surgeries had absent documentation. In the univariate analysis, compliance was associated with several clinical and procedural factors including older median age, female gender, White race, shorter procedure duration, elective procedure, outpatient procedure, and lower ASA score. Unadjusted SSI rate per 100 procedures was lower in those compliant with nasal iodine compared to noncompliant (1% and 1.6% respectively, p=0.01). (Table 1) After adjusting for age, gender, race, procedure type, and procedure duration, there was no significant difference in odds of SSI associated with nasal iodine compliance. (Odds ratio 0.78, p=0.23) (Table 2) **Conclusion:** Use of nasal iodine on day of surgery did not impact odds of SSI after adjusting for other clinical factors. This study is limited by inclusion of cases with absent documentation of nasal iodine and differences in clinical and procedural characteristics between compliant and noncompliant patients. Further studies are needed to assess effect of nasal iodine on SSI.

